# Device modeling of high performance and eco-friendly ${\text {FAMASnI}}_{3}$ based perovskite solar cell

**DOI:** 10.1038/s41598-024-66485-0

**Published:** 2024-07-04

**Authors:** Alireza Alipour, Hossein Alipour

**Affiliations:** 1 Department of Physics, Illinois Institute of Technology, Chicago, IL 60616 USA; 2 Department of Electrical Engineering, Azad University of Lahijan, Lahijan, Gilan 1616 Iran

**Keywords:** Eco-friendly perovskites, Mixed-organic-cation perovskites, Composition engineering, Interface engineering, Optoelectronic characterizations, Solar cells, Solar cells

## Abstract

Developing environmentally friendly and highly efficient inverted perovskite solar cells (PSCs) encounters significant challenges, specifically the potential toxicity and degradation of thin films in hybrid organic-inorganic photovoltaics (PV). We employed theoretical design strategies that produce hysteresis-reduced, efficient, and stable PSCs based on composition and interface engineering. The devices include a mixed-organic-cation perovskite formamidinium methylammonium tin iodide (${\text {FAMASnI}}_{3}$) as an absorber layer and zinc oxide (ZnO) together with a passivation film phenyl-C61-butyric acid methyl ester (${\text {PC}}_{61}\text {BM}$) as a double-electron transport layer (DETL). Furthermore, a nickel oxide (NiO) layer and a trap-free junction copper iodide (CuI) are used as a double-hole transport layer (DHTL). The optoelectronic characterization measurements were carried out to understand the physical mechanisms that govern the operation of the devices. The high power conversion efficiencies (PCEs) of 24.27% and 23.50% were achieved in 1D and 2D simulations, respectively. This study illustrates that composition and interface engineering enable eco-friendly perovskite solar cells, improving performance and advancing clean energy.

## Introduction

In an era where energy demand is increasing rapidly, there has been a growing tendency towards renewable energy sources such as solar energy, which presents itself as a viable alternative to the dependence on fossil fuels^1,2^. As a promising prospect in this area, perovskite solar energy devices have emerged and are increasingly being explored. They offer a cost-effective means of renewable energy conversion, thanks to their ease of the synthetic process, high absorption coefficient, and exceptional electrical properties^3^. However, the field of PVs has seen a new type of device known as organic-inorganic halide PSCs demonstrated outstanding potential in converting solar light into electricity^4–9^. This development has set the stage for many research efforts to improve the efficiency of PSCs, requiring various strategies as a multidimensional task^10–12^. These strategies have included a range of approaches such as a better understanding of charge dynamics at surface interlayers by altering various formulations for synthesizing new perovskite materials^13–16^, the creation of new device geometries^10,17,18^, combining with hybrid components^19,20^, or upgrading perovskite film-forming methods^11,21,22^. These attempts have produced successful outcomes, as the PCE of PSCs has been pushed to new levels, with some studies reporting PCE as high as 26.1%^23–25^. This exceptional level of efficiency is a result of a combination of factors such as the cost-effective and solution-processable nature of lead-based PSCs^10^, high exciton mobility^26^, high absorption coefficient^27,28^, and long diffusion lengths for electrons and holes^29,30^. Using these advanced features in organic-inorganic halide perovskite solar cells represents a significant step forward in renewable energy.

Despite the significant advances in lead-based PSCs, the practical application of these solar cells presents several technological challenges. The inherent limitations are the complicated hysteresis issues and the vexing instability under ambient and illumination conditions^31,32^. Furthermore, the environmental impacts of lead-based PSCs cannot be ignored as they are non-environmentally friendly^33–35^. In effect, achieving commercial viability for the perovskite cells remains a partially completed effort given the many challenges, particularly regarding the active materials and the architecture of the solar cells that limit the charge generation/collection processes between the perovskite absorber and the charge extracting/transporting layers^32^. Consequently, optimizing the absorber and transport layers is crucial to improving the overall performance of PSCs.

The photovoltaic device architecture of PSCs exhibits a rather intricate “n-i-p” (normal) or “p-i-n” (inverted) structure that incorporates functional layers, including a front contact material, a p-type layer, a perovskite absorber, a n-type layer, a metal contact, as well as four distinct locations for interlayers. It is crucial to emphasize that the small band gap and environmentally friendly active materials, coupled with the morphology of the interlayers and the nature of the interfaces between them, play a vital role in ensuring the PSCs are highly efficient, hysteresis-minimized, and stable^32^. The inverted PSCs have been demonstrated to reduce hysteresis effects without a proportional loss in performance significantly and improved stability when exposed to light^36^.

PSCs consist of active materials with a chemical formula of ${\text {ABX}}_{3}$. The letters A, B, and X represent an organic or inorganic monovalent cation like methyl-ammonium (${\text {CH}}_{3}{{\text {NH}}_{3}}^{+} = {\text {MA}}^{+}$), formamidinium (${\text {NH}}_{2}\text {CH} = {{\text {NH}}_{2}}^{+} = {\text {FA}}^{+}$) or Cesium (${\text {Cs}}^{+}$), a metal divalent cation such as lead (${\text {Pb}}^{2+}$) or tin (${\text {Sn}}^{2+}$), and a halide (${\text {I}}^{-}$, ${\text {Br}}^{-}$, ${\text {Cl}}^{-}$), respectively^11,22^. The lead-based PSCs have a band gap, $\text {E}_{\text{g}}$, that is larger than 1.4 eV, which hinders their PCE from reaching the theoretical limit of 33% as determined by the Shockley-Queisser limit for an $\text {E}_{\text{g}}$ of 1.34 eV^37–40^. The presence of toxic heavy metals such as Pb in these PSCs raises environmental concerns^41–45^, which can be resolved by substituting lead with elements such as germanium (Ge), bismuth (Bi), antimony (Sb), and specifically tin (Sn) which has a similar valence and ionic radius^46,47^. To overcome the toxicity issue and prepare for narrower band gaps for achieving higher PCEs, various Sn-based PSCs have been studied extensively in recent years^43,44,46–53^. In their efforts to enhance the efficacy of Sn-based PSCs, Wang et al.^54^ devised an innovative synthetic strategy to produce a high-quality methylammonium tin iodide (${\text {MASnI}}_{3}$) film resulting in an outstanding PCE of 7.78% for the perovskite cell. Liu and co-workers^55^ utilized the perovskite of formamidinium tin iodide (${\text {FASnI}}_{3}$) and derived a PCE of 13.4%.

However, despite Sn-based PSCs exhibiting superior performance compared to other Pb-free alternatives, they still fall behind Pb-based PSCs in terms of efficiency. This can be attributed to several factors, such as the sensitivity of ${\text {Sn}}^{2+}$ to oxidation, unlike ${\text {Pb}}^{2+}$, which benefits from the inert pair effect, leading to significant device degradation in ambient conditions^56,57^. The formation of Sn vacancies with low energy often results in high hole concentrations, which causes severe carrier recombination within the solar cells^58,59^. Besides, the rapid reaction between ${\text {SnI}}_{2}$ and organic ammonium salts makes it harder to control the film morphology through solution processing^60,61^. Furthermore, using the charge transport layers from Pb-based PSCs in Sn-based devices may lead to poor energy level alignment and instability, ultimately decreasing the overall performance^62^. Hence, to stabilize the perovskite phase and increase the efficiency of Sn-based PSCs, a variety of techniques has been employed, specifically, the mixing of different monovalent cations emerging as the most common method in composition engineering^16,63–67^. Zhao et al.^62^ examined the mixed-cation engineering approach and proposed the purely Sn-based composite perovskites with the structure ${\text {(FA)}}_{\text{x}}{\text {(MA)}}_{1-\text{x}}{\text {SnI}}_{3}$ ($x =$ 0.00, 0.25, 0.50, 0.75, 1.00) as the light-harvesting layer. Their efforts reached their peak when they achieved a PCE of 8.12% for the active layer ${\text {(FA)}}_{0.75}{\text {(MA)}}_{0.25}\text {SnI}_{3}$, along with an open-circuit voltage ($\text {V}_{\text {oc}}$) of 0.61 V^62^.

In the realm of solar cell architecture, there have been many suggestions for interlayer materials, with metal oxides such as ${\text {ZnO}}$ and ${\text {NiO}}$ standing out as up-and-coming options for serving as the ETL and HTL, respectively^32^. These materials exhibit exceptional charge carrier mobility, possess a wide band gap, and display favorable alignment of valence band levels to perovskite. Additionally, their ability to operate at lower processing temperatures adds to their attractiveness^31,36^. Nonetheless, the weak chemical stability, imperfect contact, ion motion, and charge recombination that arises at the interface between the ETL or HTL and the perovskite absorber are the main factors behind the hysteresis issues and instability that deteriorates the device’s performance^31,32,36^. To address these disadvantages, a technique known as interface engineering has been employed to modify the band energy offsets and enhance the intimacy of contact at these interfaces, thereby mitigating interfacial loss processes and optimizing the overall performance of PSCs^32^. The use of ${\text {ZnO}}$, as discovered by Xing et al.^68^, can result in the light soaking phenomenon and current-voltage density (J-V) hysteresis, whereas using ${\text {PC}}_{61}\text {BM}$ leads to the reduction of J-V hysteresis. Notably, Cho et al.^69^ were able to enhance the electron extraction process by inserting ${\text {PC}}_{61}\text {BM}$ between the ${\text {ZnO}}$ and the active layer, which resulted in a significant improvement of the PCE to 7.7%. Saranin et al.^36^ developed a double-hole transport configuration incorporating ${\text {CuI}}$ and ${\text {NiO}}$ interlayers. This enabled good contact and a trap-free junction for hole collection, ultimately leading to a +10% enhancement in solar cell efficiency.

In this study, we aimed to explore the theoretical foundations of Sn-based PSCs design, leveraging both the SCAPS-1D^70^ and SILVACO ATLAS-2D^71^ device simulators to guide our investigation. Specifically, we focused on the use of mixed-organic-cation films ${{\text {(FA)}}_{\text{x}}{\text {(MA)}}_{\mathrm{1-x}}{\text {SnI}}_{3}}$ in lead-free perovskite absorbers, varying the content of FA and MA cations ($x =$ 0.00, 0.25, 0.50, 0.75, 1.00) to achieve optimal performance. We implemented a DETL (${\text {ZnO}}/{\text {PC}}_{61}\text {BM}$) and a DHTL ($\text {NiO}/\text {CuI}$) configuration to improve charge extraction/transportation and reduce trap state concentrations, respectively. Additionally, ${\text {Al}}$ was utilized as a back metal contact to enhance device performance further. As part of our investigation, we examined a range of optoelectronic characterizations, including absorber thicknesses, series and shunt resistances, operating temperature, capacitance and Mott-Schottky, and generation-recombination rates, as well as J-V and quantum efficiency (QE) curves. These analyses allowed us to identify the optimal PCE of 24.27% for the device based on ${{\text {(FA)}}_{0.75}{\text {(MA)}}_{0.25}{\text {SnI}}_{3}}$, as well as a $\text {V}_{\text{oc}}$ of 1.08 V, a short-circuit current density ($\text {J}_{\text{sc}}$) of 26.69 mA/$\text {cm}^{2}$, and a fill factor (FF) of 84.30%, as determined through the use of SCAPS-1D. We further verified the performance of our best configuration through SILVACO ATLAS-2D simulation findings, which yielded a PCE of 23.50%, $\text {V}_{\text{oc}}$ of 0.95 V, $\text {J}_{\text{sc}}$ of 30.61 mA/$\text {cm}^{2}$, and FF of 81.16%. In doing so, we underscored the critical importance of implementing a double interlayer transport/extraction configuration and cation mixing as two key strategies for creating high-performance PSCs that are both environmentally friendly and effective.

## Device architecture and simulation

### Material characterization

**Figure 1:**
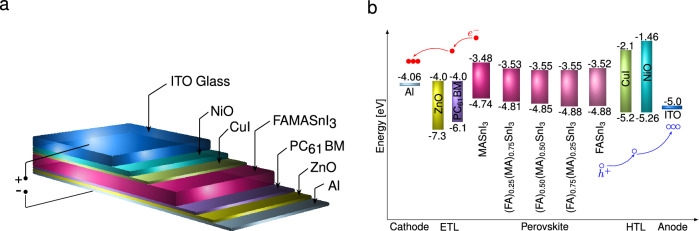
(**a**) Device design configuration of simulated “p-i-n” planar lead-free perovskite solar cell based on composition and interface engineering. (**b**) Scheme of the energy level diagram of the device.

Although our work is focused on the simulation of solid-state planar heterojunction “p-i-n” solar cells, understanding the experimental fabrication steps is crucial for accurately modeling the device. The design is based on the intricacies of composition and interface engineering, where the architecture comprises a back contact, a DETL, an active material layer, a DHTL, and a transparent conduction oxide (${\text {ITO}}$). A visual representation of the device’s structure can be seen in Fig. 1a. The process starts with cleaning a TCO substrate (like ITO) using acetone and isopropyl alcohol. A p-type ${\text {NiO}}$ layer is deposited via atomic layer deposition (ALD) or thermal evaporation, followed by an interlayer of ${\text {CuI}}$ as the second HTL using thermal evaporation or spin-coating^36^. The active layer, the mixed-organic-cation (${\text {FAMASnI}}_{3}$), is spin-coated from appropriate precursors and annealed^62^. For the ETLs, a layer of ${\text {ZnO}}$ is added using ALD or spin-coating, followed by ${\text {PC}}_{61}\text {BM}$ via spin-coating^68,69^. Finally, a thin aluminum (${\text {Al}}$) layer is deposited via thermal evaporation or sputtering as the top contact. Our simulation reproduces these steps to ensure accurate performance predictions.

### SCAPS-1D simulation methodology

The primary objective of this study is to simulate the impact of optoelectronic characterizations of planar heterostructure perovskite solar cells on the performance parameters. SCAPS-1D package version 3.3.05, a modeling tool, employs simulation calculations originating from solving a complex set of differential equations governing the intricate operations of one-dimensional semiconductor devices. Doing so provides an insight into the ideal characteristics exhibited by solar cells^72–74^. Among the key equations utilized in this simulation, the first significant relationship is Poisson’s equation. Derived from Maxwell’s equations of electromagnetism, this equation establishes a connection between the charge contained within the material and the electric field generated by the excess surplus charge. Additionally, it accounts for the electric potential created in the process. This equation, represented as Eq. (1), offers a fundamental understanding of the system’s behavior;

1 $$\begin{aligned} -\frac{d^{2}\phi }{d^{2}x}&= \frac{dE}{dx} = \frac{\rho (x)}{K\epsilon _{0}},\\ \rho (x)&= q\left[ p(x) - n(x) + N^{+}_{\text{d}}(x) - N^{-}_{\text{a}}(x) + p_{\text{t}}(x) - n_{\text{t}}(x) \right] , \end{aligned}$$

where *x*, $\phi$(*x*), E(*x*), $\rho$(*x*), K, and $\epsilon _{0}$ are the thickness, electrostatic potential, electric field, space charge density, dielectric constant, and permittivity of free space, respectively. *q*, *n*(*x*), *p*(*x*), $\text {N}^{+}_{\text{d}}$(*x*), and $\text {N}^{-}_{\text{a}}$(*x*) represent the fundamental unit of charge, the concentration of electrons and holes, and donor and acceptor ionized doping concentrations, respectively. $p_{\text{t}}$(*x*) and $n_{\text{t}}$(*x*) represent the number of trapped holes and electrons, respectively.

Introducing the current density equations, we explore the material’s detailed electron and hole transport mechanisms. These equations, the second fundamental relationship, incorporate the drift and diffusion currents. They substantially influence the material’s transport properties, shaping its behavior and characteristics. These equations, residing within Eq. (2), contribute to our understanding of the intricate interplay between charge carriers and their movement within the system:

2 $$\begin{aligned} J_{n}&= q \left[ n(x) \mu _{n} E (x) + D_{n} \frac{dn}{dx} \right] , \\ J_{p}&= q \left[ p(x) \mu _{p} E (x) - D_{p} \frac{dp}{dx} \right] , \end{aligned}$$

where $\text {J}_{{n}}$ and $\text {J}_{{p}}$ are the current density of the electron and hole, $\text {D}_{{n}}$ and $\text {D}_{{p}}$ represent electron and hole diffusion coefficients, and $\mu _{n}$ and $\mu _{p}$ are the mobility of the electron and hole, respectively. The third fundamental relationship explores the domain of continuity equations, which offer insights into the material’s intricate charge generation and recombination kinetics. These equations are provided below as Eq. (3):

3 $$\begin{aligned} \frac{dJ_{n}}{dx}&= q \left[ R_{n} - G + \frac{dn}{dt} \right] , \\ \frac{dJ_{p}}{dx}&= q \left[ G - R_{p} + \frac{dp}{dt} \right] , \end{aligned}$$

where G is the generation rate, and $\text {R}_{{n}}$ and $\text {R}_{{p}}$ denote the recombination rate for the electrons and the holes, respectively. To accurately measure the performance of the solar cell, the simulation in SCAPS-1D reproduces the conditions under standard settings. These conditions include an ambient temperature of 300 K, a frequency of 1 MHz, and the AM 1.5 G sunlight spectrum. Visualizing the energy level diagram for the materials employed in the device architecture, Fig. 1b provides an illustrative representation. The values utilized in this simulation for both the device and material parameters have been meticulously optimized. They are sourced from relevant theories, experiments, and scholarly literature^62,75–78^. A comprehensive summary of these optimized values is presented in Table 1, offering a valuable reference for the simulation and analysis process.

**Table 1:** Optimized materials proprieties of ETLs, HTLs, and active materials used in the optoelectronic simulation.

Material parameters	HTL		Active material $\left[ {{\text {(FA)}}_{\text{x}}{\text {(MA)}}_{1-{\text{x}}}{\text {SnI}}_{3}}\right]$					ETL	
	NiO	CuI	$x = 0$	$x = 0.25$	$x = 0.50$	$x = 0.75$	$x = 1$	${\text {PC}}_{61}\text {BM}$	ZnO
Thickness, *x* [nm]	50$^{\text{a}}$	50$^{\text{a}}$	850$^{\text{a}}$	850$^{\text{a}}$	850$^{\text{a}}$	850$^{\text{a}}$	850$^{\text{a}}$	50$^{\text{a}}$	50$^{\text{a}}$
Band gap, $\text {E}_{\text{g}}$ [eV]	3.8	3.1	1.26	1.28	1.30	1.33	1.36	2.1	3.3
Electron affinity, $\chi$ [eV]	1.46	2.1	3.48	3.53	3.55	3.55	3.52	4.00	4.00
Dielectric permittivity, $\varepsilon _{\text{r}}$	10.07	6.5	8.2	8.2	8.2	8.2	8.2	18	9
CB EDOS, $\text {N}_{\text{c}}$ [$\text {cm}^{-3}$]	2.8 $\times$ 10$^{19}$	2.8 $\times$ 10$^{19}$	1.0 $\times$ 10$^{18}$	1.0 $\times$ 10$^{18}$	1.0 $\times$ 10$^{18}$	1.0 $\times$ 10$^{18}$	1.0 $\times$ 10$^{18}$	2.2 $\times$ 10$^{18}$	3.7 $\times$ 10$^{18}$
VB EDOS, $\text {N}_{\text{v}}$ [$\text {cm}^{-3}$]	1.0 $\times$ 10$^{19}$	1.0 $\times$ 10$^{19}$	1.0 $\times$ 10$^{18}$	1.0 $\times$ 10$^{18}$	1.0 $\times$ 10$^{18}$	1.0 $\times$ 10$^{18}$	1.0 $\times$ 10$^{18}$	1.8 $\times$ 10$^{19}$	1.8 $\times$ 10$^{19}$
Electron thermal velocity, $\delta _{n}$ [cm/s]	1.0 $\times$ 10$^{+5}$	1.0 $\times$ 10$^{+5}$	1.0 $\times$ 10$^{+6}$	1.0 $\times$ 10$^{+6}$	1.0 $\times$ 10$^{+6}$	1.0 $\times$ 10$^{+6}$	1.0 $\times$ 10$^{+6}$	1.0 $\times$ 10$^{+7}$	1.0 $\times$ 10$^{+7}$
Hole thermal velocity, $\delta _{p}$ [cm/s]	1.0 $\times$ 10$^{+7}$	1.0 $\times$ 10$^{+7}$	1.0 $\times$ 10$^{+7}$	1.0 $\times$ 10$^{+7}$	1.0 $\times$ 10$^{+7}$	1.0 $\times$ 10$^{+7}$	1.0 $\times$ 10$^{+7}$	1.0 $\times$ 10$^{+5}$	1.0 $\times$ 10$^{+5}$
Electron mobility, $\mu _{n}$ [$\text {cm}^{2}$/Vs]	1.2 $\times$ 10$^{1}$	1 $\times$ 10$^{2}$	1.6 $\times$ 10$^{0}$	2.2 $\times$ 10$^{1}$	2.2 $\times$ 10$^{1}$	2.2 $\times$ 10$^{1}$	2.2 $\times$ 10$^{1}$	2.0 $\times$ 10$^{-3}$	1.0 $\times$ 10$^{2}$
Hole mobility, $\mu _{p}$ [$\text {cm}^{2}/$Vs]	2.8 $\times$ 10$^{0}$	4.39 $\times$ 10$^{1}$	1.6 $\times$ 10$^{0}$	2.2 $\times$ 10$^{1}$	2.2 $\times$ 10$^{1}$	2.2 $\times$ 10$^{1}$	2.2 $\times$ 10$^{1}$	2.0 $\times$ 10$^{-3}$	2.5 $\times$ 10$^{1}$
Shallow donor density, $\text {N}_{\text{D}}$ [$\text {cm}^{-3}$]	0	0	0	0	0	0	0	1.0 $\times$ 10$^{21}$	1.0 $\times$ 10$^{18}$
Shallow acceptor density, $\text {N}_{\text{A}}$ [$\text {cm}^{-3}$]	1.0 $\times$ 10$^{18}$	1.0 $\times$ 10$^{18}$	1.0 $\times$ 10$^{14}$	1.0 $\times$ 10$^{14}$	1.0 $\times$ 10$^{14}$	1.0 $\times$ 10$^{14}$	1.0 $\times$ 10$^{14}$	0	0
Defect density, $\text {N}_{\text{t}}$ [$\text {cm}^{-3}$]	1.0 $\times$ 10$^{15}$	1.0 $\times$ 10$^{15}$	3.3 $\times$ 10$^{13}$	2.5 $\times$ 10$^{13}$	2.5 $\times$ 10$^{13}$	2.5 $\times$ 10$^{13}$	3.0 $\times$ 10$^{13}$	1.0 $\times$ 10$^{15}$	1.0 $\times$ 10$^{15}$
References	^75^	^75^	^62,76^	^62^	^62^	^62^	^62,77^	^78^	^75^

^a^In this study.

### SILVACO ATLAS-2D simulation methodology

The SILVACO ATLAS version 5.28.1.R was used to model the highest efficient inverted perovskite solar cell with configuration ${\text {Al}}/{\text {ZnO}}/{\text {PC}}_{61}\text {BM}/{{\text {(FA)}}_{0.75}{\text {(MA)}}_{0.25}{\text {SnI}}_{3}}/{\text {CuI}}/{\text {NiO}}/ {\text {ITO}}$ in two dimensions (2D) as a secondary verification of the device’s performance. The ATLAS is a physical-based device simulator that models the transport of carriers through a device structure by approximating the device operation onto a 2D grid and applying a set of differential equations derived from Maxwell’s laws [2D version of the Eqs. (1), (2), and (3)] to this grid^71^. In this simulation, the thickness of the cell is 1000 nm and 1050 nm in the X and Y directions, respectively. Moreover, we have defined the meshing process uniformly on the X-axis and non-uniformly on the Y-axis. This method makes the size of triangles in the mesh grid smaller and leads to more accurate results.

To investigate the optical and electrical interactions in the device structure, the effects of scattering mechanisms and carrier recombination process were considered using two physical models: the mobility model and the recombination model. The constant low-field mobilities, electron $\mu _{n}$, and hole $\mu _{p}$ mobilities are used for each layer as a default model independent of doping concentration, carrier densities, and electric field and account for lattice scattering due to temperature^71^. Shockley-Read-Hall (SRH) and the Auger (AUGER) recombination models were considered for the ETL layers, which are needed to consider the effects of defects (or traps) that cause states in the band gap as well as third-order recombination process^79,80^. For active material, optical (OPTR) and SRH recombination models were chosen to consider the effects of the band-band recombination process and trap^79,80^. The SRH model is also considered for HTL layers to have the effect of defects in the performance of the devices. In this simulation, same as the SCAPS-1D model, the corresponding material proprieties used for each layer are presented in Table 1. Table 2 shows two physical models employed for each layer in the ATLAS-2D simulation.

**Table 2:** Material type, mobility model, and recombination model for each layer in the ATLAS-2D simulation methodology.

Layer	Material type	Mobility model	Recombination model
ETL	Inorganic	Constant low-field	Auger (AUGER) & Shockley-Read-Hall (SRH)
PSC	Organic-inorganic	Constant low-field	Optical recombination (OPTR) & Shockley-Read-Hall (SRH)
HTL	Inorganic	Constant low-field	Shockley-Read-Hall (SRH)

## Results and discussion

### SCAPS-1D simulation results

#### Effect of interfacial engineering and band diagram

**Figure 2:**
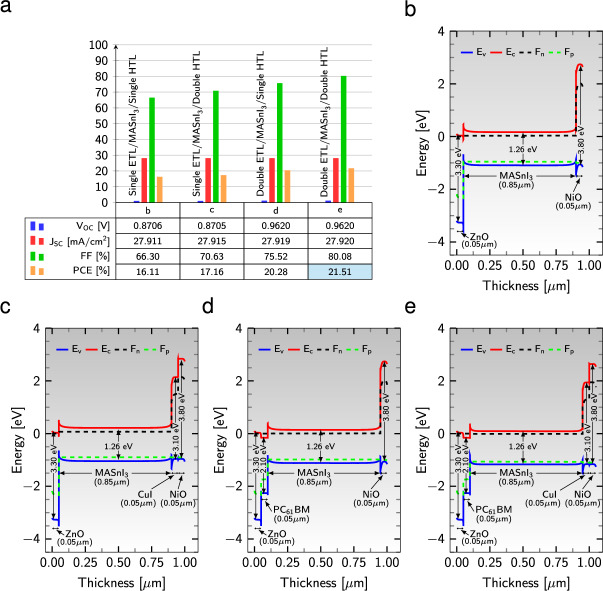
(**a**) Variation of performance parameters of ${\text {MASnI}_{3}}$ absorber layer-based PSCs for four PSC structures from left to right: ${\text {ZnO}}/{\text {MASnI}}_{3}/{\text {NiO}}, {\text {ZnO}}/{\text {MASnI}}_{3}/{\text {CuI}}/{\text {NiO}}$, ${\text {ZnO}}/{\text {PC}}_{61}\text {BM}/{\text {MASnI}}_{3}/{\text {NiO}}$, and ${\text {ZnO}}/{\text {PBC}}_{61}{\text {BM}}/{\text {MASnI}}_{3}/{\text {CuI}}/{\text {NiO}}$. (**b**)–(**e**) Energy band diagram of the four device configurations with different ETLs and HTLs. ${\text {Al}}$ is considered as a back metal contact in all simulations.

The configuration of an inverted PSC device involves the utilization of an HTL to collect holes from the active material and transfer them to the transparent conduction layer. Conversely, an ETL facilitates the extraction of electrons from the light-absorbing layer, prevents their recombination with the holes in the absorber layer, and directs them toward the back metal contact. However, the interfaces between the active material and the charge-extracting/transporting layers present challenges due to a high concentration of charge trapping and poor chemical compatibility. These factors significantly influence the flow of charges and the stability of the devices. This study simulated five different device configurations using SCAPS-1D to optimize the performance. The performance parameters of these configurations, while varying the HTLs and ETLs, are depicted in Fig. 2a.

A visual representation of the interface engineering reveals that the device configuration ${\text {Al}}/{\text {ZnO}}/{\text {PC}}_{61}\text {BM}/{\text {MASnI}}_{3}/{\text {CuI}}/{\text {NiO}}$ yields the best performance, with a PCE of 21.51%, FF of 80.08%, $\text {J}_{\text{sc}}$ of 27.92 mA/$\text {cm}^{2}$, and $\text {V}_{\text{oc}}$ of 0.962 V.

The energy band diagrams, depicting various interlayers with the same active material, are displayed in Fig. 2b–e. The movement of photogenerated carriers is influenced by the Conduction Band Offset (CBO) and Valence Band Offset (VBO) between the active material and interlayers. Figure 2b shows that the CBO between the ${\text {MASnI}}_{3}$ absorber layer and the ${\text {NiO}}$ HTL is significantly large, effectively blocking electron motion from the absorber to the front contact ${\text {ITO}}$. Similarly, the large VBO between ${\text {MASnI}}_{3}$ and ${\text {ZnO}}$ inhibits hole transport from the active material to the back electrode ${\text {Al}}$. Introducing an additional HTL, ${\text {CuI}}$, between ${\text {MASnI}}_{3}$ and ${\text {NiO}}$ (see Fig. 2c), as well as sandwiching a second ETL, ${\text {PC}}_{61}\text {BM}$, between ${\text {MASnI}}_{3}$ and ${\text {ZnO}}$ (see Fig. 2d), creates a double barrier, effectively blocking electron and hole flows. Figure 2e demonstrates that employing a DHTL and a DETL in the PSC structures results in the suppression of the Fermi energy levels of holes (${\text {F}}_{\text{p}}$) and electrons (${\text {F}}_{\text{n}}$) towards the valence band (${\text {E}}_{\text{v}}$) and conduction band (${\text {E}}_{\text{c}}$), respectively. The appropriate work function of the ${\text {Al}}$ cathode, which is nearly aligned with the conduction band of the ${\text {ZnO}}$ ETL, corrects the Schottky barrier at the interface. This alignment improves electron flow towards the cathode and consequently boosts the PCE. Moreover, the suitable work function of the ${\text {NiO}}$ anode and the valence band of the ${\text {ITO}}$ suppresses the ohmic contact at the interface, facilitating hole transport and further enhancing the PCE. In general, it has been observed that device efficiency is enhanced when a DETL and a DHTL are simultaneously employed in interfacial engineering. This improvement can be attributed to establishing good contact and trap-free junctions between the perovskite and interlayers, facilitating efficient charge collection at the interface^36,69^.

#### Effect of composition engineering and band diagram

**Figure 3:**
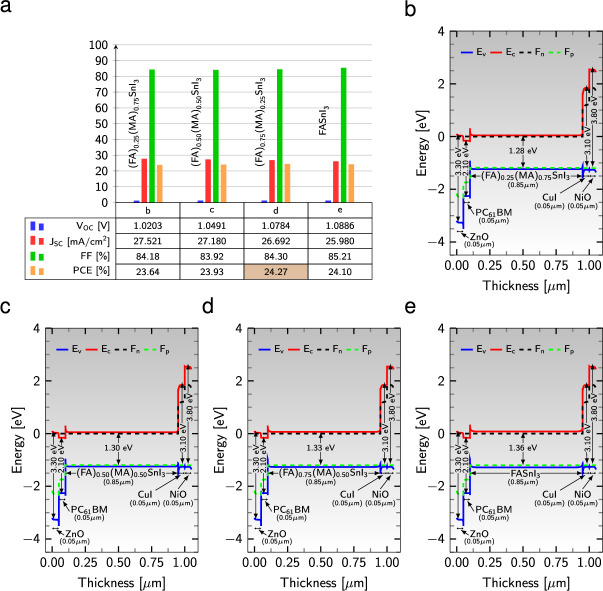
(**a**) Variation of performance parameters of four PSC structures including different cation mixing perovskites from left to right: ${{\text {(FA)}}_{0.25}{\text {(MA)}}_{0.75}{\text {SnI}}_{3}}$, ${{\text {(FA)}}_{0.50}{\text {(MA)}}_{0.50}{\text {SnI}}_{3}}$, ${{\text {(FA)}}_{0.75}{\text {(MA)}}_{0.25}{\text {SnI}}_{3}}$, and ${{\text {FASnI}}_{3}}$ with keeping additional ETLs and HTLs. (**b**)–(**e**) Energy band diagram of the four device configurations with different active materials and the same ETLs and HTLs. ${\text {Al}}$ is considered as a back metal contact in all simulations.

The intricate interplay between active materials and their band gap energies holds the key to optimizing the performance of solar cells. By carefully selecting absorber layers with band gaps that align with the photon-rich region of the solar spectrum, we can fine-tune the properties of perovskites. An innovative approach known as multicomponent engineering offers vast potential in this regard. This approach involves combining different organic-inorganic cations, resulting in profound effects on the optical properties, photovoltaic efficiency, and stability of perovskites^80,81^. To investigate the performance characteristics, we simulated three distinct types of mixed-organic-cation perovskites: ${{\text {(FA)}}_{0.25}{\text {(MA)}}_{0.75}{\text {SnI}}_{3}}$, ${{\text {(FA)}}_{0.50}{\text {(MA)}}_{0.50}{\text {SnI}}_{3}}$, and ${{\text {(FA)}}_{0.75}{\text {(MA)}}_{0.25}{\text {SnI}}_{3}}$. Additionally, we included a single-organic-cation perovskite, namely ${{\text {FASnI}}_{3}}$, in our study. Figure 3a displays the performance parameters of these device configurations while keeping the DHTL, DETL, and ${\text {Al}}$ as the back metal contact.

A visual analysis of the composition engineering reveals that the device architecture featuring the absorber layer ${{\text {(FA)}}_{0.75}{\text {(MA)}}_{0.25}{\text {SnI}}_{3}}$ exhibits optimal performance, boasting an impressive PCE of 24.27%, a FF of 84.30%, a $\text {J}_{\text{sc}}$ of 26.69 mA/$\text {cm}^{2}$, and a $\text {V}_{\text{oc}}$ of 1.08 V. Remarkably, the efficiency of the ${{\text {FASnI}}_{3}}$-based solar cell is better than that of the ${\text {MASnI}}_{3}$-based counterpart^49^. Examining the energy band diagrams of various active materials with identical interlayers, as shown in Fig. 3b–e, we observe a notable suppression of $\text {F}_{\text{p}}$ and $\text {F}_{\text{n}}$ towards $\text {E}_{\text{v}}$ and $\text {E}_{\text{c}}$ in the double-organic-cation perovskites. This, in turn, leads to superior band alignments and, ultimately, higher efficiency compared to single-organic-inorganic cation absorbers (see Fig. 3e). Consequently, composition engineering centered around double-organic-cation perovskites yields remarkable enhancements in device performance, owing to improved perovskite film morphology and the suppression of charge carrier recombination within the devices^62,80^.

#### Effect of absorber thickness on cell performance

**Figure 4:**
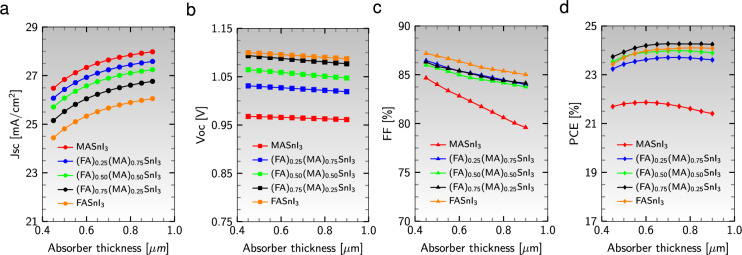
Performance parameters: (**a**) Short-circuit current density ($\text {J}_{\text{sc}}$), (**b**) open-circuit voltage ($\text {V}_{\text{oc}}$), (**c**) fill factor (FF), and (**d**) power conversion efficiency (PCE) as a function of absorber layer thickness, keeping the thickness of ETLs and HTLs fixed.

The photovoltaic parameters, including $\text {J}_{\text{sc}}$, $\text {V}_{\text{oc}}$, FF, and PCE, are significantly influenced by the thickness of the active material, which plays a crucial role in generating photo-excited charge carriers. To analyze this effect, the thickness of the absorber layers in an eco-friendly stable structure (${\text {Al}}/{\text {ZnO}}/{\text {PC}}_{61}\text {BM}/$an active material$/{\text {CuI}}/{\text {NiO}}/{\text {ITO}}$) was varied from 450 to 900 nm while keeping other parameters constant (refer to Table 1). Figure 4 shows the impact of absorber layer thickness on the performance parameters of different active materials.

As depicted in Fig. 4a, $\text {J}_{\text{sc}}$ increases for all absorber layers up to 900 nm. The thickness of 900 nm yields the highest $\text {J}_{\text{sc}}$ values of 27.98 mA/$\text {cm}^{2}$ for ${\text {MASnI}}_{3}$ and the lowest of 26.05 mA/$\text {cm}^{2}$ for ${\text {FASnI}}_{3}$. This observation suggests that increasing the cation MA content in the active materials leads to a higher absorption coefficient, enhances electron mobility, and increases the electron-hole pair generation rate compared to cation FA. Figure 4b illustrates a decreasing trend in $\text {V}_{\text{oc}}$ values as the absorber thickness increases from 450 to 900 nm. Furthermore, perovskites with higher MA contents exhibit a more pronounced decrease in $\text {V}_{\text{oc}}$, indicating an enhanced charge carrier recombination rate and larger saturation current relative to photocurrent in these perovskites. The fill factor FF gradually decreases with increasing absorber thickness, as shown in Fig. 4c. This decrement is more prominent in perovskites with higher MA contents due to increased series resistance and internal power depletion within the PSCs.

The variations in PCE can be explained by considering the competition between photon absorption rate and charge carrier recombination rate. Figure 4d reveals that the absorber layer ${\text {MASnI}}_{3}$ achieves its highest PCE (21.87%) at 600 nm and experiences a rapid drop with further thickness increase. This decline is attributed to the dominance of carrier recombination rate over photon absorption rate in thick absorber layers, resulting in decreased overall device performance. Furthermore, as the FA content in the active materials increases, an intriguing observation emerges: the PCE exhibits an initial increase and peaks at 700 nm for the active material ${{\text {(FA)}}_{0.75}{\text {(MA)}}_{0.25}{\text {SnI}}_{3}}$, reaching a maximum value of 24.27%. This PCE remains constant within the thickness range of 700 to 900 nm. The underlying reason for this variation can be attributed to a delicate equilibrium between light absorption and carrier transport in mixed-organic-cation perovskite absorbers. This equilibrium facilitates a greater number of charge carriers to reach the charge-collecting electrodes, leading to a substantial increase in overall PCE. Additionally, the influence of varying the thicknesses of HTLs and ETLs (ranging from 20 to 120 nm) was investigated. The results (not shown here) indicate no significant change in the performance parameters under these thickness variations.

#### Effect of parasitic losses on cell performance

**Figure 5:**
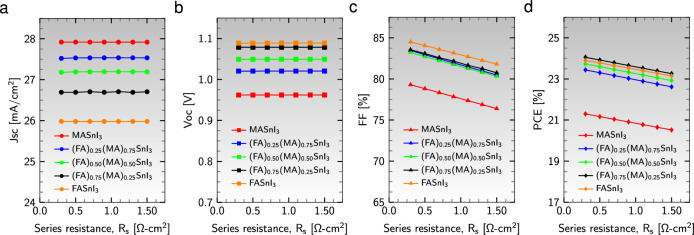
Effect of series resistance, $\text {R}_{\text{s}}$, on performance parameters (**a**) $\text {J}_{\text{sc}}$, (**b**) $\text {V}_{\text{oc}}$, (**c**) FF, and (**d**) PCE for different absorber layers.

The performance of solar cells is significantly influenced by the series ($\text {R}_{\text{s}}$) and shunt ($\text {R}_{\text{sh}}$) resistances. The presence of $\text {R}_{\text{s}}$ can be attributed to the contact resistance of the metallic electrode and the ohmic resistance of the transparent electrode^82^. Figure 5 shows the effect of varying $\text {R}_{\text{s}}$ (from 0.3 to 1.5 $\Omega$ $\text {cm}^{2}$) while keeping $\text {R}_{\text{sh}}$ constant at $10^{5}$ $\Omega$ $\text {cm}^{2}$ on the photovoltaic properties of five perovskite cells (${\text {Al}}/{\text {ZnO}}/{\text {PC}}_{61}\text {BM}/$an active material$/{\text {CuI}}/{\text {NiO}}/{\text {ITO}}$).

During the adjustment of $\text {R}_{\text{s}}$, the parameters $\text {J}_{\text{sc}}$ and $\text {V}_{\text{oc}}$ remain unchanged, indicating that the performance parameters are unaffected by variations in $\text {R}_{\text{s}}$. At a fixed $\text {R}_{\text{s}}$, we observed a decrease in $\text {J}_{\text{sc}}$ and an increase in $\text {V}_{\text{oc}}$ with increasing FA content in the absorber layers (see Fig. 5a and b). The FF and PCE decrease as $\text {R}_{\text{s}}$ increase, leading to leakage currents in all device configurations associated with absorbers. This decrement in FF and PCE is particularly notable in active materials with higher MA content at a constant $\text {R}_{\text{s}}$ (see Fig. 5c and d). Moreover, it is important to highlight that the absorber ${{\text {(FA)}}_{0.75}{\text {(MA)}}_{0.25}{\text {SnI}}_{3}}$ exhibits the most favorable performance in decreasing PCE with increasing $\text {R}_{\text{s}}$.

**Figure 6:**
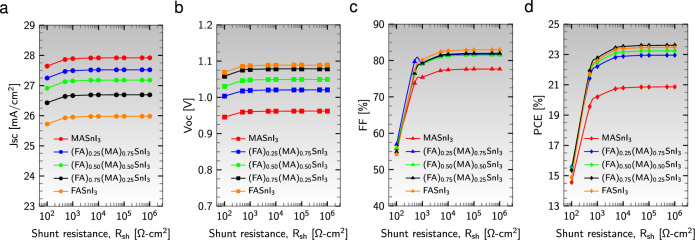
Effect of shunt resistance, $\text {R}_{\text{sh}}$, on performance parameters (**a**) $\text {J}_{\text{sc}}$, (**b**) $\text {V}_{\text{oc}}$, (**c**) FF, and (**d**) PCE for different absorber layers.

The $\text {R}_{\text{sh}}$ is mainly influenced by various alternative charge recombination processes, such as defects on the device surface and leakage currents across device edges. Figure 6 illustrates the impact of varying $\text {R}_{\text{sh}}$ (from $10^{2}$ to $10^{6}$ $\Omega$ $\text {cm}^{2}$) on the performance parameters of the five optimized solar cell structures, with $\text {R}_{\text{s}}$ being constant at 1 $\Omega$ $\text {cm}^{2}$.

All performance parameters exhibit a similar trend with variations in $\text {R}_{\text{sh}}$. Initially, the $\text {J}_{\text{sc}}$, $\text {V}_{\text{oc}}$, FF, and PCE values increase rapidly from $10^{2}$ to $10^{4}$ $\Omega$ $\text {cm}^{2}$ and then remain constant as $\text {R}_{\text{sh}}$ continues to increase. Figure 6 demonstrates that active materials with higher FA contents display lower values of $\text {J}_{\text{sc}}$ but higher values of $\text {V}_{\text{oc}}$, FF, and PCE. Based on the above description, reducing $\text {R}_{\text{s}}$ and increasing $\text {R}_{\text{sh}}$ may greatly enhance the stability and efficiency of photovoltaic solar cells, even under low illumination conditions. Therefore, optimizing parasitic resistances through compositional engineering can reduce losses, enhancing the performance and reliability of perovskite solar cells.

#### Effect of working temperature

Exploring the influence of working temperature on device performance reveals many complex phenomena occurring in solar panels. While simulations were conducted at the standard ambient temperature of 300 K, it is crucial to acknowledge the outdoor installation of solar panels, where the intricate interplay of thermal instability among layers impacts overall device stability and longevity^83^. A fixed lighting intensity of 1000 W/m^2^ was maintained to assess the impact of operating temperature, while the working temperature was systematically varied from 290 to 360 K, holding all optimized parameters constant.

**Figure 7:**
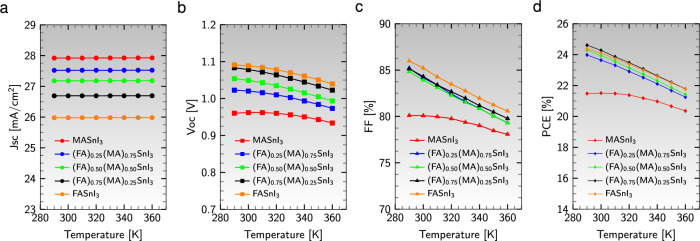
Variation of performance parameters of the device configuration ${\text {Al}}/{\text {ZnO}}/{\text {PC}}_{61}\text {BM}/$an active material$/{\text {CuI}}/{\text {NiO}}/{\text {ITO}}$ with temperature.

Figure 7a reveals that $\text {J}_{\text{sc}}$ exhibits remarkable stability across all device structures despite temperature fluctuations. The slight current variation can be attributed to the minor effects of band gap reduction induced by temperature increase^83^. Notably, the active layer ${\text {MASnI}}_{3}$ shines with a magnificent $\text {J}_{\text{sc}}$ value of 27.92 mA/$\text {cm}^{2}$, while the absorber ${\text {FASnI}}_{3}$ exhibits a more modest $\text {J}_{\text{sc}}$ of 25.98 mA/$\text {cm}^{2}$ as the temperature rises. In contrast, the reversed saturation current density $\text {J}_{\text{0}}$ experiences a notable jump with increasing temperature, leading to decreased $\text {V}_{\text{oc}}$ value across all device configurations (see Fig. 7b). Equation (4), derived under the assumption of $\text {R}_{\text{sh}} \gg$ $\text {R}_{\text{s}}$ at the open-circuit state, elegantly captures the intricate relationship between $\text {J}_{\text{0}}$ and $\text {V}_{\text{oc}}$:

4 $$\begin{aligned} V_{\text{oc}} = \frac{nK_{B}T}{q} ln \left[ 1 + \frac{J_{sc}}{J_{0}} \right] , \end{aligned}$$

Here, *n* denotes the ideality factor, *q* symbolizes the charge of an electron, and $\text {K}_{{B}}$ represents the Boltzmann constant. Figure 7c and d reveal the compelling decline of FF and PCE values as the temperature increases in all solar cell devices. This decrement can be attributed to the escalation of defects, the intricate deformation stress experienced by diverse layers, and the expansion of diffusion distances that occur with temperature rise^83^. Notably, the PSC based on ${{\text {(FA)}}_{0.75}{\text {(MA)}}_{0.25}{\text {SnI}}_{3}}$ emerges as a robust competitor, showcasing the least decline in PCE among cells with mixed cations, thereby maintaining an acceptable level of performance despite temperature variations.

#### Effect of capacitance and Mott-Schottky

**Figure 8:**
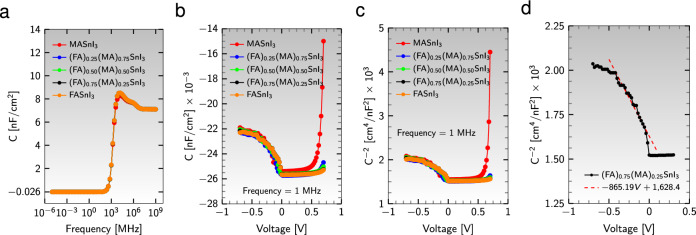
(**a**) Capacitance per unit area, C, versus frequency, (**b**) C as a function of bias voltage, and (**c**) Mott-Schottky ($\text {C}^{-2}$) plot for different active layers. (**d**) One way to determine the acceptor density, $\text {N}_{\text{A}}$, and the built-in potential, $\text {V}_{\text{bi}}$, of the active material ${\text {(FA)}{0.75}\text {(MA)}{0.25}\text {SnI}_{3}}$ is by analyzing the slope of a linear fit on the MS plot and examining the intercept.

The capacitance-voltage measurements (CV) analysis can distinguish various physical effects occurring on different timescales within a device. For instance, the transport of free carriers occurs on a shorter timescale (higher frequency) compared to trapping and de-trapping due to space charge effects, which happen on a longer timescale (lower frequency)^84,85^. At low frequencies, the capacitance increases due to slow traps and ionic charges, whereas the recombination of charge carriers decreases the capacitance^84–86^. The decrement in capacitance can even become negative due to many reasons, such as accumulation and migration of ions at the interface of charge extraction layers, trapping and de-trapping of charge carriers, frequency-induced changes in resistance, electrochemical reactions^87^, as well as non-ohmic behavior at one of the extracting contacts^88^. Figure 8a shows that the capacitance value is negative up to the frequency $10^2$ MHz for the five perovskite absorber layers. By increasing the frequency to $10^4$ MHz, capacitance reaches 8.5 nF/$\text {cm}^{2}$ from the approximate value of $-0.026$ nF/$\text {cm}^{2}$. However, this value decreases again for frequencies higher than $10^{4}$ MHz, but it is positive.

The Mott-Schottky (MS) analysis based on CV measurements is a technique that examines the space charge distributions formed by a junction capacitance and how the junction capacitance varies with applied reverse bias^89^. The capacitance per unit area (C) can be determined as a function of the applied bias voltage ($\text {V}_{{bias}}$) using Eq. (5);

5 $$\begin{aligned} C = \left[ \frac{q \epsilon _{r} N_{A}}{2(V_{bi} - V_{bias})} \right] ^{1/2}, \end{aligned}$$

where *q* is the elementary charge, $\text {N}_{\text{A}}$ is the acceptor density, $\text {V}_{\text{bi}}$ is the built-in potential, and $\epsilon _{r}$ is the dielectric permittivity of the active material. Moreover, Fig. 8b indicates that the value of the negative capacitance increased as the voltage increased from $-0.7$ to 0 V, remained constant until 0.5 V, and an exponentially increased pattern was observed after 0.5 V. The MS plot (see Fig. 8c) shows that capacitance changes parabolically with the applied voltage, given by the Eq. (6);

6 $$\begin{aligned} C^{-2} = \left( \frac{-2}{q \epsilon _{r} N_{A}}\right) V_{bias} + \frac{2 V_{bi}}{q \epsilon _{r} N_{A}}, \end{aligned}$$

with the slope of a linear fit on the MS plot providing information about $\text {N}_{\text{A}}$ and the intercept indicating $\text {V}_{\text{bi}}$ (see Fig. 8d). Table 3 tabulated the $\text {N}_{\text{A}}$ and $\text {V}_{\text{bi}}$ of different active materials in the device configuration ${\text {Al}}/{\text {ZnO}}/{\text {PC}}_{61}\text {BM}/$an active material$/{\text {CuI}}/{\text {NiO}}/{\text {ITO}}$. The increased values of acceptor concentration and built-in voltage in active materials with higher FA contents indicate changes in the electrical properties of the material, such as an increase in the number of available charge carriers and a larger potential barrier for charge carriers, respectively.

**Table 3:** The acceptor density, $\text {N}_{\text{A}}$, and built-in potential, $\text {V}_{\text{bi}}$, of different active materials in the device configuration ${\text {Al}}/{\text {ZnO}}/{\text {PC}}_{61}\text {BM}/$an active material$/{\text {CuI}}/{\text {NiO}}/{\text {ITO}}$.

Active material	$\text {N}_{{A}}$ [$\text {cm}^{-3}$]	$\text {V}_{{bi}}$ [V]
${\text {MASnI}}_{3}$	$1.28 \times 10^{15}$	1.28
${{\text {(FA)}}_{0.25}{\text {(MA)}}_{0.75}{\text {SnI}}_{3}}$	$1.34 \times 10^{15}$	1.39
${{\text {(FA)}}_{0.50}{\text {(MA)}}_{0.50}{\text {SnI}}_{3}}$	$1.54 \times 10^{15}$	1.60
${{\text {(FA)}}_{0.75}{\text {(MA)}}_{0.25}{\text {SnI}}_{3}}$	$1.76 \times 10^{15}$	1.88
${\text {FASnI}}_{3}$	$1.77 \times 10^{15}$	1.89

#### Effect of generation and recombination rate

**Figure 9:**
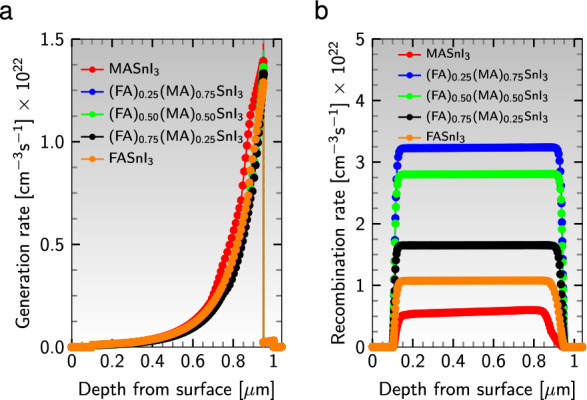
(**a**) Generation rate and (**b**) recombination rate for different active layers in the device configuration ${\text {Al}}/{\text {ZnO}}/{\text {PC}}_{61}\text {BM}/$an active material$/{\text {CuI}}/{\text {NiO}}/{\text {ITO}}$.

When a light pulse hits a solar cell device, high-energy photons propel electrons from the valence band to the conduction band of the absorber layer, creating electron-hole pairs in the carrier generation process^90^. As shown in Fig. 9a, the generation rate peaks at the absorber-HTL interface and is greater for absorbers with more MA cations.

On the other hand, recombination occurs when excess carriers pair up and recombine, resulting in an increased saturation current density that decreases the current that can be collected and reduces the energy conversion efficiency^90^. As shown in Fig. 9b, single cation absorbers have the least recombination rate, while the active layer ${{\text {(FA)}}_{0.75}{\text {(MA)}}_{0.25}{\text {SnI}}_{3}}$ has the lowest recombination rate among the cation-mixing absorbers.

#### J-V and QE characteristics

**Figure 10:**
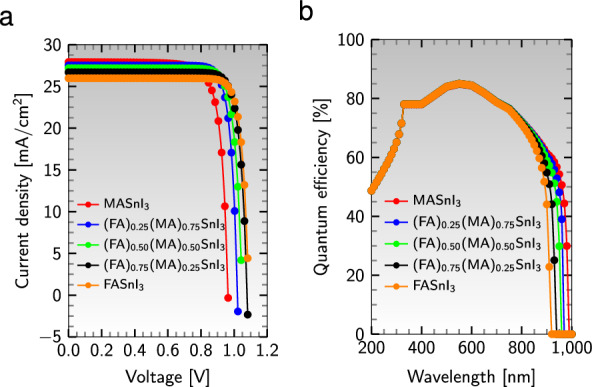
(**a**) J-V characteristics and (**b**) QE curve of different absorbers in the device configuration ${\text {Al}}/{\text {ZnO}}/{\text {PC}}_{61}\text {BM}/$an active material$/{\text {CuI}}/{\text {NiO}}/{\text {ITO}}$.

In the realm of solar cell performance analysis, one of the critical factors is the illuminated photocurrent density-voltage (J-V) characteristic, which provides crucial information on the main parameters utilized to evaluate the cell’s efficiency. Figure 10a depicts the optimal J-V curves for various perovskite absorbers in the cell’s configuration ${\text {Al}}/{\text {ZnO}}/{\text {PC}}_{61}\text {BM}/$an active material$/{\text {CuI}}/{\text {NiO}}/{\text {ITO}}$. From this graph, one can infer that perovskites that comprise a higher proportion of MA have a greater current density but a lower voltage, while the converse holds for absorbers that contain more FA. The area under the J-V curve proves that the absorber ${{\text {(FA)}}_{0.75}{\text {(MA)}}_{0.25}{\text {SnI}}_{3}}$ achieves the highest efficiency among the absorber layers.

The external quantum efficiency (QE), the ratio of photogenerated charge carriers to the number of photons that hit the cell’s surface, is a vital parameter for solar cell efficiency assessment. The QE is a function of the incident light’s wavelength, $\lambda$, and is measured by exposing the cell to monochromatic light and then calculating the photocurrent, $\text {I}_{\text{ph}}$, through the device^90^. Nevertheless, due to optical and electrical losses such as parasitic absorption and recombination, the QE of most solar cells is less than 100%. As depicted in Fig. 10b, the QE curves vary for different absorbers to different wavelengths, and all absorber layers exhibit a maximum QE of 85% at the green wavelength of 550 nm. The maximum QE value for the absorbers remains relatively constant. Still, the range of wavelengths for light absorption decreases with increasing FA contents, indicating that most of the absorption happens close to the visible wavelength range of 380 to 700 nm.

#### Effect of defect density and doping concentration

**Figure 11:**
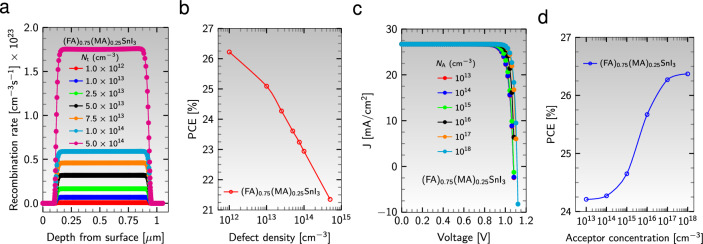
(**a**) Variation of recombination rate with depth from the surface for different defect densities and (**b**) PCE as a function of defect density. (**c**) J-V curve for various acceptor doping concentrations and (**d**) PCE versus acceptor doping concentrations.

In the pursuit of high-performance PSCs, the quality and morphology of the active layer must be considered, as point defects such as lattice vacancy, interstitial, Schottky, and Frenkel defects in absorbers are unavoidable due to reduced film features^91^. The presence of defects leads to the recombination of charge carriers by trap states, making the Shockley-Read-Hall (SRH) recombination the dominant type in the absorber layer^91,92^. To examine the effect of absorber defect density, $\text {N}_{\text{t}}$, on device performance, the SRH recombination model was utilized to investigate the impact of $\text {N}_{\text{t}}$ on the recombination rate. Specifically, the active layer ${{\text {(FA)}}_{0.75}{\text {(MA)}}_{0.25}{\text {SnI}}_{3}}$ was studied in the solar cell configuration ${\text {Al}}/{\text {ZnO}}/{\text {PC}}_{61}\text {BM}/$an active material$/{\text {CuI}}/{\text {NiO}}/{\text {ITO}}$, which demonstrated the highest efficiency in the previous sections. Figure 11a displays the variation of recombination rate versus depth from the surface for various values of $\text {N}_{\text{t}}$, revealing that as the defect concentration increases from $10^{12}$ to $5\times 10^{14}$ $\text {cm}^{-3}$, the recombination rate increases significantly. This leads to a decrease in the diffusion length of charge carriers and a drastic reduction of PCE from 26.22 to 21.35% (see Fig. 11b).

In addition to the absorber defect density, the acceptor density of holes in the absorber layer significantly impacts photovoltaic performance. When the Sn-based device is exposed to air, the oxidation of ${{\text {Sn}}^{2+}}$ to ${{\text {Sn}}^{4+}}$ has been shown to ruin the photovoltaic performance. However, this issue can be addressed by increasing the acceptor doping concentration^91^. To study the effect of the acceptor doping concentration, $\text {N}_{\text{A}}$, of the absorber, the acceptor density of the ${{\text {(FA)}}_{0.75}{\text {(MA)}}_{0.25}{\text {SnI}}_{3}}$ layer in the same cell’s configuration was varied from $10^{13}$ to $10^{18}$ $\text {cm}^{-3}$. Figure 11c shows the variation of J-V characteristics and PCE to the acceptor doping concentration of the perovskite layer. The area under the J-V graph expands as the acceptor doping concentration increases, indicating an increase in PCE from 24.21 to 26.37%, as depicted in Fig. 11d. This may be due to the increase in the generation rate of carriers and the decrease in the Fermi energy level of the hole, leading to a simultaneous increase in current and voltage^91^. Furthermore, increasing acceptor doping concentration increases the electric field at the heterostructure interfaces, enhancing the charge separation mechanism and, consequently, PCE^77,91^.

#### Optimum values for device parameters

After thoroughly examining the various factors that influence the performance parameters, particularly the effect of temperature, we have arrived at a resolute conclusion. It has been determined that the absorber thickness, defect density, and acceptor doping concentration should be fine-tuned to achieve the optimized values of 850 nm, $2.5\times 10^{13}$ $\text {cm}^{-3}$, and $1\times 10^{14}$ $\text {cm}^{-3}$, respectively, as illustrated in the comprehensive Table 1. This precise selection process ensured that the resulting photovoltaic devices demonstrated a reliable PCE across all analyzed device configurations.

### ATLAS-2D simulation results

#### Optoelectronic characteristics

**Figure 12:**
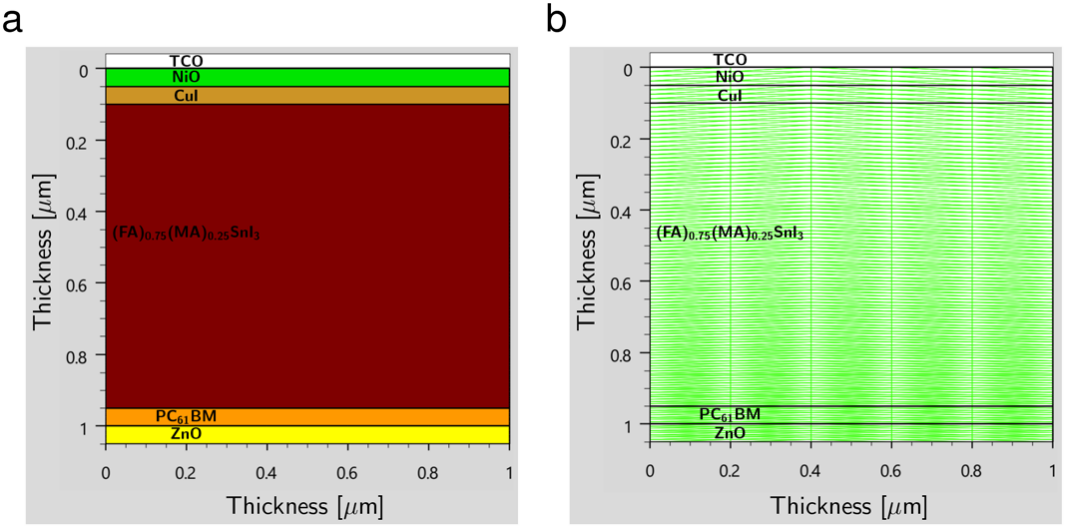
Schematic (**a**) device and (**b**) meshed structures with vertical 2D heterojunction used for simulation in ATLAS.

In the scope of optical characteristics, we explore the intricacies of the highest efficient planar 2D heterostructure ${\text {Al}}/{\text {ZnO}}/{\text {PC}}_{61}\text {BM}/{{\text {(FA)}}_{0.75}{\text {(MA)}}_{0.25}{\text {SnI}}_{3}}/{\text {CuI}}/{\text {NiO}}/ {\text {ITO}}$. The 2D device and its meshed configuration are illustrated in Fig. 12.

**Figure 13:**
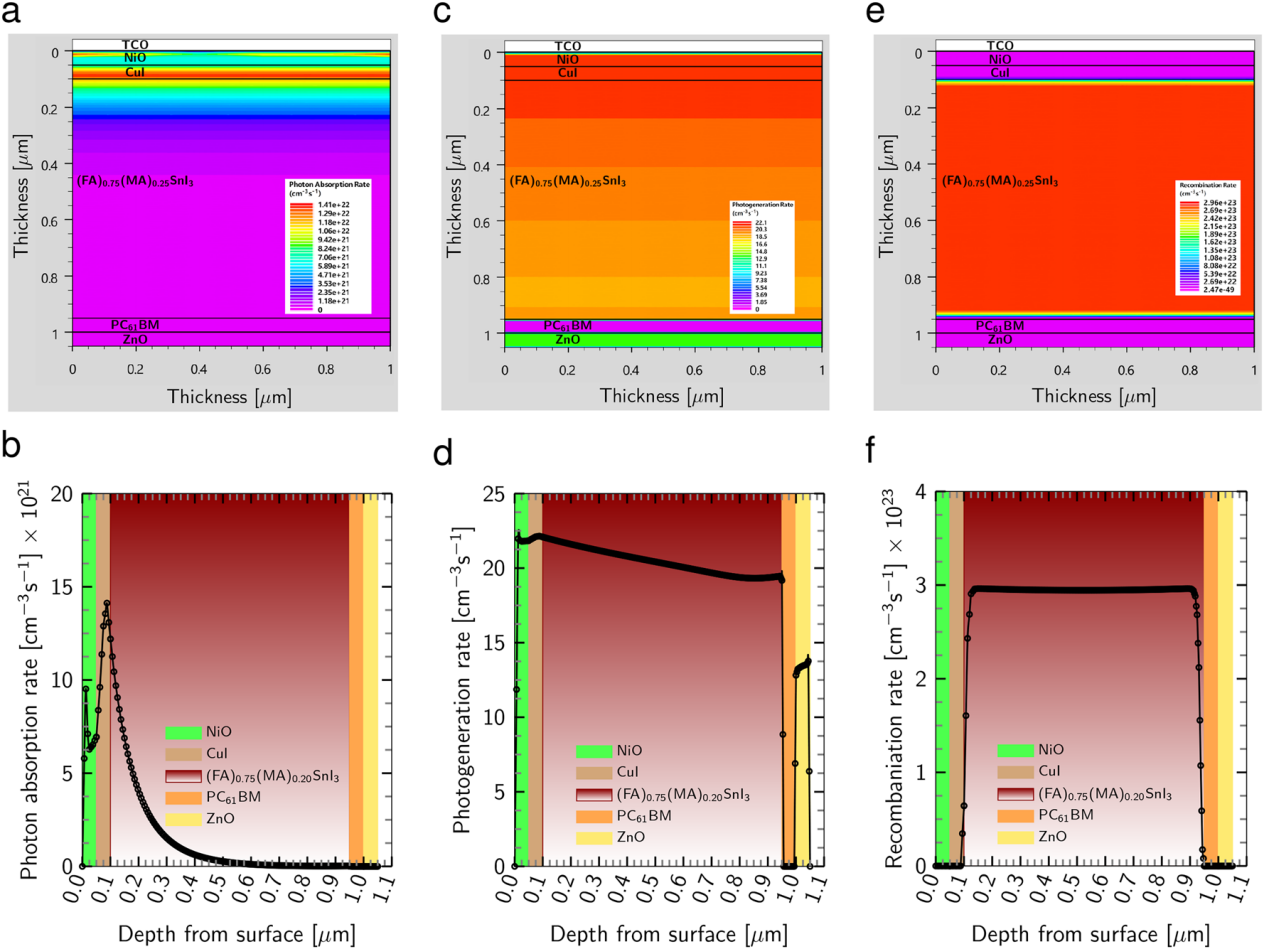
(**a**) Schematic photon absorption rate and (**b**) simulated photon absorption rate curve across the device. (**c**) Schematic photogeneration rate and (**d**) simulated photogeneration rate curve. (**e**) Schematic recombination rate and (**f**) simulated recombination rate curve across the PSC.

As the light penetrates PSC, the absorption rate increases from HTLs to the absorber, reaching its maximum value of 1.41 $\times 10^{22}$ ${\text {cm}}^{-3} {\text{s}}^{-1}$ in the interface between the active layer and ${\text {CuI}}$ HTL, as depicted in Fig. 13a and b. However, as we study the PSC, the absorption rate starts to decrease exponentially at a depth of 450 nm due to the physical and optical properties of the absorber, such as the high thickness and the semi-transparency, which prevents light from penetrating downward.

**Figure 14:**
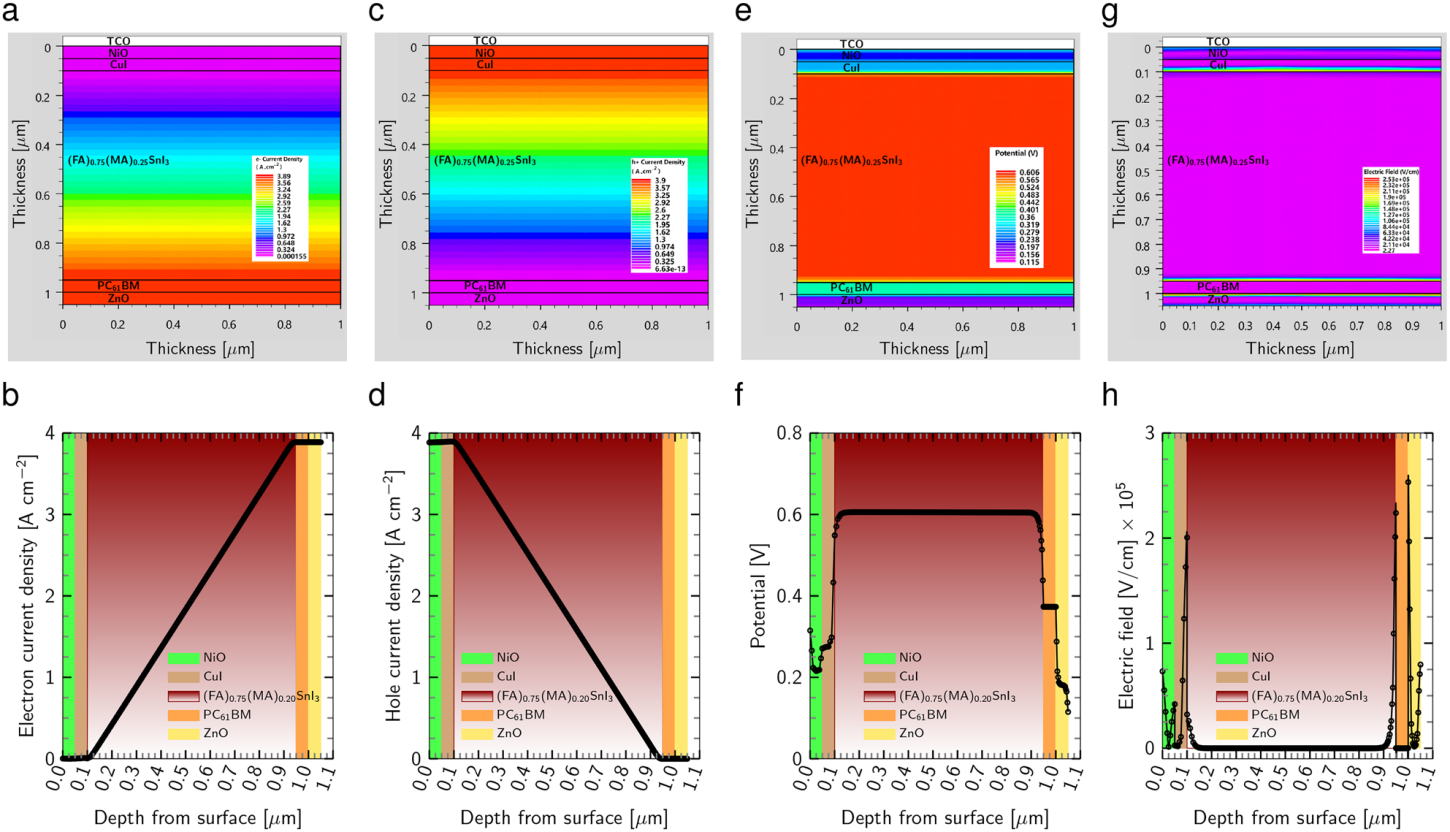
(**a**) Schematic electron current density and (**b**) simulated electron current density curve across the whole device. (**c**) Schematic hole current density and (**d**) simulated hole current density curve. (**e**) Schematic potential and (**f**) simulated potential curve in the PSC. (**g**) schematic electric field and (**h**) simulated electric field curve across the PSC.

Figure 13c and d reveal that the HTLs and perovskite/HTLs interface exhibit the highest photogeneration rate (22.1 ${\text{cm}}^{-3} {\text{s}}^{-1}$) as a result of the high light absorption rate around these areas, leading to the generation of more electron-hole pairs. In addition, the ${\text {PC}}_{61}\text {BM}$ ETL passivates slow ion migration, thereby minimizing the J-V hysteresis in the structure, promoting the light soaking process, and consequently increasing the photogeneration rate in the ${\text {ZnO}}$ ETL^68^. Figure 13e and f show that the recombination rate is the highest inside the perovskite layer and at the interface between this layer and the carrier transport layers, posing a challenge for maintaining a high photogeneration rate. To explore the current density of electrons and holes, we turn to Fig. 14a–d. It is discovered that the maximum electron and hole current densities of 3.9 A/$\text {cm}^{2}$ are related to ETLs and HTLs layers, respectively, indicating that the charge carrier extraction and transportation process is exceptionally efficient through HTLs and ETLs.

Furthermore, we observe the PSC’s electrical potential, represented in Fig. 14e and f. Due to the high photo absorption rate in HTLs, the potential rises in these layers and reaches a maximum value of 0.606 V inside the absorber before decreasing in the ETLs. Lastly, a strong electric field is seen in the interfaces between the absorber and interlayers, as well as between ${\text {PC}}_{61}\text {BM}$ and ${\text {ZnO}}$ (see Fig. 14g and h), as a result of the high donor/acceptor doping concentration in the interfaces^77,91^.

### 2D vs. 1D measurements

**Figure 15:**
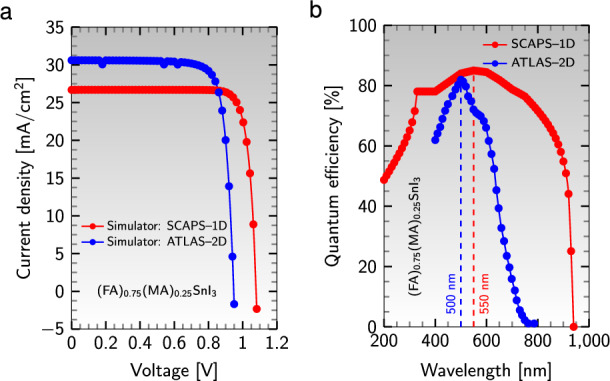
Simulated (**a**) J-V curves and (**b**) QE curves of the device structure ${\text {Al}}/{\text {ZnO}}/{\text {PC}}_{61}\text {BM}/{{\text {(FA)}}_{0.75}{\text {(MA)}}_{0.25}{\text {SnI}}_{3}}/{\text {CuI}}/{\text {NiO}}/{\text {ITO}}$ in 1D and 2D simulations.

The 1D and 2D simulated J-V curves of the device structure ${\text {Al}}/{\text {ZnO}}/{\text {PC}}_{61}\text {BM}/{{\text {(FA)}}_{0.75}{\text {(MA)}}_{0.25}{\text {SnI}}_{3}}/{\text {CuI}}/{\text {NiO}}/{\text {ITO}}$ have been displayed in Fig. 15a. It is important to note that the 2D model has a higher short circuit current than the 1D model, but its open circuit voltage is lower than that of the 1D model. However, the area under the J-V graphs is almost the same, resulting in good agreement between the two models in simulating the device.

**Figure 16:**
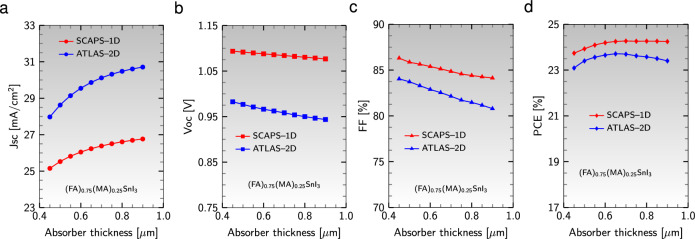
Effect of absorber thickness on (**a**) $\text {J}_{\text{sc}}$, (**b**) $\text {V}_{\text{oc}}$, (**c**) FF, and (**d**) PCE for the device’s configuration ${\text {Al}}/{\text {ZnO}}/{\text {PC}}_{61}\text {BM}/{{\text {(FA)}}_{0.75}{\text {(MA)}}_{0.25}{\text {SnI}}_{3}}/{\text {CuI}}/{\text {NiO}}/{\text {ITO}}$ in the 1D and 2D models.

The maximum value of QE (82%) has been obtained at the wavelength 500 nm for 2D simulation, while for 1D simulation, this value was 85% at the wavelength 550 nm (see Fig. 15b). Thus, we can conclude that the best QE can be achieved in the wavelength range of 500–550 nm, commonly known as the green light region. The impact of absorber thickness on the device’s configuration’s performance was examined using 1D and 2D simulations (see Fig. 16). Interestingly, almost all performance parameters exhibit the same trends as the thickness increases in 1D and 2D simulations. The maximum value of PCE (23.70%) has been achieved at 650 nm for 2D simulation, whereas for 1D simulation, this value was 24.27% at the thickness of 850 nm. As a result, we can infer that the most optimal PCE can be achieved in the thickness range of 650–850 nm.

## Simulation results vs. previous works

This section compares our proposed solar cell’s 1D and 2D simulation results with other experimental and theoretical studies to evaluate its efficiency and stability (see Table 4).

**Table 4:** The comparison of performance parameters of the presented device configuration with recently published optimum configurations.

Device architecture	Study	$\text {J}_{\text{sc}}$ [mA/$\text {cm}^{2}$]	$\text {V}_{\text{oc}}$ [V]	FF [%]	PCE [%]	Ref.
Normal 1D: ${\text {FTO}}/{\text {PC}}_{61}\text {BM}/{\text {MASnI}}_{3}/{\text {CuI}}/{\text {Au}}$	Theoretical	34.27	1.06	69.23	25.05	Jayan et al.^76^
Normal 1D: ${\text {FTO}}/{\text {Zn}}({{\text {O}}_{0.3}}, {{\text {S}}_{0.7}})/{\text {FASnI}}_{3}/{\text {CuSCN}}/{\text {Au}}$	Theoretical	28.12	1.09	84.96	25.94	Tara et al.^93^
Inverted: ${\text {ITO}}/{\text {PEDOT}}:{\text {PSS}}/{\text {FASnI}}_{3}/{{\text {C}}_{60}}/{\text {BCP}}/{\text {Al}}$	Experimental	21.95	0.64	72.50	10.16	Meng et al.^94^
Inverted 2D: ${\text {ITO}}/{\text {NiO}}/\text {p}-{{\text {MAPbI}}_{3}}/\text {n}-{{\text {MAPbI}}_{3}}/{\text {ZnO}}/{\text {Al}}$	Theoretical	20.18	1.09	86.57	19.10	He et al.^95^
Inverted: ${\text {FTO}}/{\text {NiO}}/{\text {CuI}}/{{\text {MAPbI}}_{3}}/{\text {PC}}_{61}\text {BM}/{\text {PCB}}/{\text {Al}}$	Experimental	20.60	1.07	69.00	15.26	Saranin et al.^36^
Inverted: ${\text {ITO}}/{\text {PEDOT}}:{\text {PSS}}/{{\text {(FA)}}_{0.75}{\text {(MA)}}_{0.25}{\text {SnI}}_{3}}/{{\text {C}}_60}/ {\text {BCP}}/{\text {Al}}$	Experimental	21.20	0.61	62.70	8.12	Zhao et al.^62^
Inverted 1D: ${\text {Al}}/{\text {ZnO}}/{\text {PC}}_{61}\text {BM}/{{\text {(FA)}}_{0.75}{\text {(MA)}}_{0.25}{\text {SnI}}_{3}}/{\text {CuI}}/ {\text {NiO}}/{\text {ITO}}$	Theoretical	26.69	1.08	84.30	24.27	This work
Inverted 2D: ${\text {Al}}/{\text {ZnO}}/{\text {PC}}_{61}\text {BM}/{{\text {(FA)}}_{0.75}{\text {(MA)}}_{0.25}{\text {SnI}}_{3}}/{\text {CuI}}/{\text {NiO}}/{\text {ITO}}$	Theoretical	30.61	0.95	81.16	23.50	This work

It is worth mentioning that although lead-free PSCs with single-cation absorbers like ${\text {MASnI}}_{3}$ and ${\text {FASnI}}_{3}$ have demonstrated slightly high efficiency, our lead-free solar cell with cation-mixing absorber, i.e., ${{\text {(FA)}}_{0.75}{\text {(MA)}}_{0.25}{\text {SnI}}_{3}}$, has exhibited higher PCE and specifically better stability. Moreover, the lead-based solar cell device structures with a single cation absorber, i.e., ${{\text {MAPbI}}_{3}}$, have shown less PCE, lower stability, and a harmful environmental impact. Our studied absorber has superior optical properties and similar physical properties to the absorber used in the Zhao et al.^62^ study. Furthermore, the distinct characteristics of our ETLs and HTLs compared to their interlayers have resulted in a higher PCE for our device configuration. Based on the comparisons, we can conclude that the compositional engineering (i.e., cation mixing absorber) and interface engineering (i.e., DETL/DHTL) techniques have worked excellently, thereby justifying the potential of our proposed eco-friendly high-performance PSC as a potential alternative to conventional perovskite solar cells.

## Conclusion

This study comprehensively optimized lead-free Sn-based perovskite solar cells to develop environmentally sustainable, high-performance devices. Through detailed investigations of ${\text {MASnI}}_{3}$-based solar cell structure, SCAPS-1D simulations identified the ${\text {Al}}/{\text {ZnO}}/{\text {PC}}_{61}\text {BM}/{\text {MASnI}}_{3}/{\text {CuI}}/{\text {NiO}}$ configuration as the best performer using the interface engineering, achieving a PCE of 21.51%, FF of 80.08%, $\text {J}_{\text{sc}}$ of 27.92 mA/$\text {cm}^2$, and $\text {V}_{\text{oc}}$ of 0.962 V. Further optimization using compositional engineering found that the ${{\text {(FA)}}_{0.75}{\text {(MA)}}_{0.25}{\text {SnI}}3}$ absorber layer provided the highest performance with a PCE of 24.27%, FF of 84.30%, $\text {J}_{\text{sc}}$ of 26.69 mA/$\text {cm}^2$, and $\text {V}_{\text{oc}}$ of 1.08 V. Various factors, such as absorber layer thickness, operating temperature, and parasitic resistances, capacitance, and Mott-Schottky, generation and recombination rate, and J-V and QE characteristics, were also analyzed for their impact on photovoltaic parameters. 2D theoretical analysis using the SILVACO ATLAS simulator validated the optimal structure, confirming a PCE of 23.50%, FF of 81.16%, $\text {J}_{\text{sc}}$ of 30.61 mA/$\text {cm}^2$, and $\text {V}_{\text{oc}}$ of 0.95 V. These findings demonstrate that combining compositional and interface engineering effectively enhances the efficiency and stability of Sn-based PSCs. Our research suggests that these techniques can develop high-performance, environmentally friendly, cost-effective solar cells, contributing to renewable energy solutions. Future work will include 3D simulations and fabrication of the proposed PSC to validate our optimized solar cell designs further.

## Data Availability

The datasets used and/or analyzed during the current study are available from the corresponding author upon reasonable request.
